# Supplementary material for: Dynamic Changes in Fecal Microbial Communities of Neonatal Dairy Calves by Aging and Diarrhea
Source: Animals (Basel). 2021 Apr 13;11(4):1113. doi: 10.3390/ani11041113 (PMC8070554; doi:10.3390/ani11041113)
Supplement: Supplementary file 1 [file animals-11-01113-s001.pdf]

Supple. Table 1. Chemical composition (% DM) and intake (mean ± standard deviation) of calf starter and mixed hay fed to calves.

	**Calf starter**	**Mixed hay** [1]
Chemical composition (% DM)		
DM	87.0	85.4
CP	20.3	7.73
EE	5.53	1.23
CF	6.63	32.7
Ash	6.06	5.97
NDF	-	60.0
ADF	-	33.5
Total intake (mean± SD)		
1~4 weeks	2771 ± 54.4	465 ± 10.4
5~6 weeks	3360 ± 50.6	487 ± 12.4
7~8 weeks	4075 ± 43.1	506 ± 10.8

[1]Mixed hay consisted 50% orchard grass and 50% tall fescue; Mineral premix supplied the following nutrients per kg mixed feed: vitamin A, 4200 IU; vitamin D, 7433; vitamin E, 121 U; $CuSO_4 5H_2O$, 85.7 mg; zinc oxide, 55 mg; $MnO_2 H_2O$, 55 mg; MgO, 1.88g; DM, Dry matter; CP, Crude protein; EE, Ether extract; CF, Crude fiber; NDF, neutral detergent fiber; and ADF, acid detergent fiber
